# Ensuring Quality by a Clinical Audit of the Mode of Birth: The Use of the Robson Classification System

**DOI:** 10.7759/cureus.89502

**Published:** 2025-08-06

**Authors:** Paraskevi Giaxi, Maria Dagla, Maria Iliadou, Ermioni Palaska, Athina Diamanti, Angeliki Bolou, Kleanthi Gourounti

**Affiliations:** 1 Department of Midwifery, University of West Attica, Athens, GRC

**Keywords:** birth, cesarean section, clinical audit, greece, pregnancy, quality, robson classification

## Abstract

Background

The overuse of cesarean section (CS) leads to risks in maternal and neonatal health. One of the highest rates of CS in Europe is observed in Greece, making it essential to understand the factors contributing to this elevated rate, especially among low-risk groups. The implementation of the Robson classification as a clinical audit tool is a key step in enhancing the quality of obstetric and midwifery care, allowing for the systematic monitoring and evaluation of the mode of delivery. The present study aimed to identify the clinical and demographic predictors of CS in women classified in Robson 1 and 2a categories, based on deliveries conducted in 2019 at a private hospital in Athens, Greece.

Methodology

A retrospective analysis was conducted employing data from 8,572 deliveries in 2019 at a private hospital in Athens by classifying women based on the Robson classification. Multiple logistic regression analysis was performed to identify significant predictors in the two groups.

Results

For the Robson 1 category, the significant predictors were smoking during pregnancy (odds ratio (OR) = 1.75, p = 0.001), use of in vitro fertilization (IVF) (OR = 2.22, p = 0.002), presence of pre-existing maternal conditions (OR = 1.50, p = 0.014), and fetal-related pregnancy pathology (OR = 3.90, p = 0.013). In addition, maternal age between 20 and 29 years was associated with significantly lower odds of CS compared to those aged 30-39 years (OR = 0.65, p=0.022), while gestational age of 39+0 to 41+6 weeks compared to 37+0 to 38+6 weeks (OR = 0.60, p<0.0001) was also linked to lower risk. For the Robson 2a category, the significant predictors were Greek nationality (OR = 1.91, p < 0.0005), smoking during pregnancy (OR = 1.91, p < 0.0005), IVF conception (OR = 1.51, p = 0.044), and neonatal birth weight ≥4,000 g (OR = 2.95, p < 0.0005). On the contrary, reduced odds were noted for maternal age between 20 and 29 years in comparison to the 30-39-year group (OR = 0.70, p = 0.005). The presence of gestational diabetes (OR = 0.66, p = 0.019) and neonatal birth weight below 3,000 g (OR = 0.73, p = 0.027) were also protective factors.

Conclusions

The use of the Robson classification as a clinical audit tool facilitates the identification of clinical practice patterns associated with the increased CS rates, contributing to the targeted improvement of perinatal quality of care.

## Introduction

Clinical audit consists of a structured quality assurance mechanism, targeted to improve the quality of healthcare through the systematic evaluation of clinical practices against established standards. Clinical audit involves assessing structures, processes, and outcomes, which is crucial to identify gaps, implement changes, and monitor potential improvements [1]. In midwifery, it is an important tool to improve the quality of care and maternal outcomes through the evaluation of clinical practices against evidence-based standards, leading to targeted interventions and improvements in care delivery [2].

Cesarean section (CS) has led to a significant reduction in the risk of morbidity and mortality in the face of serious obstetric conditions; however, its overuse has led to a “cesarean epidemic” [3]. This problem has been widely recognized by the World Health Organization (WHO), which supports that although CS can be lifesaving when medically indicated, unnecessary CS might lead to significant short- and long-term risks for mothers and their babies. To address this problem, the WHO recommends a wide range of interventions, such as educational programs and clinical audits [4]. The Robson classification system is used to categorize and analyze data in clinical audit cycles to reduce CS rates [5]. In addition, the evidence regarding the need to reduce CS rates is continually growing owing to the increasing recognition of negative outcomes after CS. In a recent Swedish study with a large sample (N = 2,442,330), CS was associated with increased risk (hazard ratio = 1.21) for lymphoblastic leukemia during childhood [6].

During the last decades, high rates of CS have been reported in Europe. Despite the need to reduce CS rates, no de-escalation is observed in Europe [7]. In a relevant study, Amyx et al. [8] found that during 2015-2019, the rates of CS increased in 12 out of 28 countries, remained steady in seven countries, and decreased in nine countries. They also found that CS for the Robson 2a category increased, making it essential to further investigate this category. Interestingly, Amyx et al. [8] could not find data from Greece, although they supported that it was the country with the highest prevalence at the beginning of their study period, at about 50%. A further analysis by Wladimiroff et al. [9] confirmed these observations, reporting that in 2023 the prevalence of CS in Greece was 48.6%.

The year 2019 is an interesting turning point to draw conclusions for CS and, in general, for midwifery, as it was the last year before the pandemic, which led to an increase in CS following induction of labor [10]. Taking the year 2019 as a final time point, in a previous study, we found an extremely high prevalence of CS (60.9%) in a private hospital in Greece. We also noted that women in the Robson 1 and 2a categories had high rates of CS at 38.7% and 32.7%, respectively [11]. Yet, this might indicate an overuse of CS, especially for the Robson 1 category. For example, in a study from Canada, Gu et al. [12] noted that although 33.5% of Robson 2a women had undergone CS, the prevalence for Robson 1 women was much lower, at 18.4%. Based on the aforementioned data, in this study, we aimed to identify the drivers of CS in the women included in our original study, belonging to Robson 1 and 2a categories. Based on the aforementioned data, this study aimed to identify the clinical and demographic drivers of CS among women included in our original 2019 [11] study who were classified in Robson 1 and 2a categories.

## Materials and methods

In this retrospective, cross-sectional study, the sample was recruited from a private hospital in Athens, Greece. This hospital conducts approximately 10,000 deliveries per year, handling all types of pregnancies, including high-risk pregnancies. The hospital is located in the capital city, but is also visited by women living in rural Greece, as private hospitals are mostly concentrated in the big cities of the country. According to the Hellenic Statistical Authority, a total of 83,756 deliveries were performed in Greece during 2019 [13], leading to the conclusion that the studied hospital serves a considerable proportion of all women giving birth in Greece. We selected 2019 as the last complete year before the COVID‑19 pandemic to avoid pandemic‑related changes in obstetric practice.

The data collection process was initiated after receiving approval from the hospital’s ethics committee (approval number: 1146/24-09-2020). As the women had already signed a GDPR form, receiving written consent from the women was not required. The inclusion criteria for the present study were the following: (1) women with gestational ages ≥22 weeks, and (2) birth weights ≥500 g. Stillborn fetuses/neonates and women with no access to their medical records were excluded. Hence, from a total of 8,681 deliveries, 73 were excluded due to stillborn fetuses/neonates, and 36 were excluded because there was no access to their medical records, resulting in a total of 8,572 cases that were further analyzed. Because the study encompassed all eligible births in that year, no a priori power or sample size calculation was performed; the available data determined the sample size.

Data were extracted manually from the hospital’s electronic medical records by a trained data collector using a standardized data collection form developed for the study. The following data were retrieved: age, smoking status during pregnancy, use of assisted reproduction technology, obstetric history (parity and previous CS), onset of labor (spontaneous/induced/CS before labor), fetal lie or presentation (cephalic, breech, transverse, or oblique), number of fetuses (single or multiple), gestational age (weeks), mode of birth (vaginal birth, operative vaginal birth, or CS), and indicator for CS. Pre-existing conditions were defined as chronic medical diagnoses established before conception (such as chronic hypertension, type 1 or type 2 diabetes mellitus, thyroid disorders, cardiovascular disease, asthma, and other chronic illnesses recorded in the medical file). Women with at least one of these diagnoses were coded as having a pre‑existing condition. Smoking status was recorded dichotomously as smoker versus non‑smoker based on self‑reported cigarette use at any point during pregnancy. Information on the number of cigarettes smoked per day was not available, which we acknowledge as a limitation. The following data were obtained from the neonatal medical records: the neonate’s sex (boy or girl) and birth weight. Missing values were fewer than 3% for all variables and were addressed by excluding incomplete cases from the respective analyses (case-wise deletion). The births were classified according to the Robson classification, as described by the WHO [14].

The data analysis was performed using SPSS version 21 for Windows (IBM Corp., Armonk, NY, USA). At first, descriptive statistics were performed, using absolute values and proportions, in case of categorical data, and means as well as standard deviations, for quantitative data. Furthermore, multiple logistic regression was used using all the sociodemographic and clinical data as independent variables with mode of delivery (CS vs. vaginal birth) as the dependent variable. Variable selection was based on existing literature and clinical relevance. Two different models were developed, one for women belonging in the Robson 1 category and one for women belonging in the Robson 2a category. All assumptions for the application of logistic regression (including homoscedasticity, linearity, normal distribution, and independence of residuals, as well as multicollinearity among independent variables) were tested, and no violations were identified in either category. The p-value was set at 0.05 for all analyses.

## Results

The demographic characteristics of the women are presented in Table 1. In total, 928 (10.8%) women out of the 8,572 women who gave birth during the study period were in category 1, with a mean age of 33.01 years (SD = 4.6 years). The majority of the sample, 656 (70.7%), belonged to the age group 30-39 years. For 741 (79.8%) women, the place of residence was Athens/Attica, and 878 (94.6%) women reported Greece as their place of birth. Most participants (534, 57.5%) gave birth between 39+0 days and 41+6 days of gestation. CS deliveries accounted for 359 (38.7%) of births, while 569 (61.3%) births were vaginal deliveries, of which 394 (42.0%) were normal vaginal births and 179 (19.3%) were instrumental vaginal deliveries. Further, 223 (24%) women reported having a pre-existing medical condition. More specifically, 50 (5.4%) mothers reported a pathological condition during pregnancy, of which 30 (3.2%) were of maternal origin and 20 (2.2%) of fetal origin. Additionally, 121 (13%) women had gestational diabetes, with 19 (2.0%) receiving insulin treatment and 101 (10.9%) managing it through diet. During pregnancy, 251 (27.0%) women smoked, and 82 (8.8%) women had undergone assisted reproductive techniques.

**Table 1:** Demographic and clinical characteristics of the study population. Data are presented as n (%) or mean ± SD, as appropriate. No statistical comparisons were performed. SD = standard deviation; IVF = in vitro fertilization

		Robson 1		Robson 2a	
		Ν	%	Ν	%
Age (years)	Mean ± SD	33.01 ± 4.60 (17–50)		31.97 ± 4.55 (17–46)	
	<20	1	0.1	9	0.5
	20–29	201	21.7	463	27.1
	30–39	656	70.7	1,165	68.2
	≥40	70	7.5	72	4.2
Place of residence	Athens/Attica	741	79.8	1,360	79.6
	Other regions of Greece	187	20.2	345	20.2
	Outside Greece	0	0	4	0.2
Nationality	Other	50	5.4	74	4.3
	Greek	878	94.6	1,635	95.7
Gestational age (weeks)	37+0–38+6	394	42.4	442	25.9
	39+0–41+6	534	57.5	1,266	74.1
	≥42	0	0	1	0.1
Type of birth	Vaginal birth	390	42.0	793	46.4
	Operative vaginal birth	179	19.3	358	20.9
	Cesarean section	359	38.7	558	32.7
Pre-existing maternal condition	No	705	76	1,318	77.1
	Yes	223	24.0	391	22.9
Pregnancy pathology	No	878	94.6	1,653	96.7
	Yes	50	5.4	56	3.3
Pregnancy pathology (maternal cause)	Yes	30	3.2	44	2.6
Pregnancy pathology (fetal cause)	Yes	20	2.2	12	0.7
Gestational diabetes	No	807	87.0	1,508	88.2
	Yes	121	13.0	201	11.8
	Diet-controlled	101	10.9	158	9.3
	Insulin-treated	19	2.0	43	2.5
Assisted reproduction	No	846	91.2	1,592	93.2
	Yes	82	8.8	117	6.8
Smoking	No	677	73.0	1,217	71.2
	Yes	251	27.0	492	28.8

Overall, 1,709 women were in category 2a, with a mean age of 31.9 years (SD = 4.55 years). The majority of the sample, 1,165 (68.2%) women, belonged to the age group of 30-39 years. For most of the sample, 1,360 (79.6%), the place of residence was Athens/Attica, and 1,635 (95.7%) reported Greece as their place of birth. Furthermore, 1,266 (74.1%) women gave birth between 39+0 days and 41+6 days of gestation, while only one (0.1%) mother gave birth at ≥42 weeks of gestation. CS deliveries accounted for 558 (32.7%) of births, while 1.151 (67.3%) of births were vaginal deliveries, of which 793 (46.4%) were normal vaginal births and 358 (20.9%) were operative vaginal deliveries. Moreover, 391 (22.9%) women reported having a pre-existing medical condition. Specifically, 56 (3.3%) mothers reported a pathological condition during pregnancy, of which 44 (2.6%) were of maternal origin and 12 (0.7%) of fetal origin. Additionally, 201 (11.8%) women had gestational diabetes, with 43 (2.5%) receiving insulin treatment and 151 (9.3%) managing it through diet. During pregnancy, 492 (28.8%) women smoked, and 117 (6.8%) of the women had undergone assisted reproductive techniques.

The results of the multiple logistic regression analysis to identify factors associated with CS versus vaginal birth for women classified in the Robson category 1 are presented in Table 2. According to the analysis presented in the table, significant predictors included maternal age (p = 0.024 and p = 0.022), smoking (p = 0.001), in vitro fertilization (IVF) conception (p = 0.002), gestational age (p < 0.0001), pre-existing conditions (p = 0.014), and pathological fetal pregnancy (p = 0.013), all of which statistically significantly affected the likelihood of participants undergoing a CS. Women aged 20-29 years had a 35% lower likelihood of giving birth by CS. Women who smoked had a 75% higher likelihood of giving birth by CS. Women with assisted pregnancies had a 2.2 times higher likelihood of giving birth by CS. Women between 39+0 and 41+6 weeks of gestation had a 40% lower likelihood of giving birth by CS compared to those at 37+0 and 38+6 weeks of gestation. Women with pre-existing conditions had a 50% higher likelihood of giving birth by CS. Women with pathological fetal pregnancies had a 3.9 times higher likelihood of giving birth by CS.

**Table 2:** Multiple logistic regression for Robson category 1. Dependent variable: cesarean versus vaginal birth. P-values were derived from logistic regression. OR = odds ratio; CI = confidence interval

Variable	OR	95% CI	P-value
Age			0.024
20–29 years	0.65	0.44–0.94	0.022
30–39 years	1.00	-	-
>40 years	1.40	0.79–2.46	0.250
Nationality			0.094
Other	1.00	-	
Greek	0.59	0.32–1.09	
Smoking			0.001
No	1.00	-	
Yes	1.75	1.24–2.46	
In vitro fertilization			0.002
No	1.00	-	
Yes	2.22	1.34–3.67	
Gestational age (weeks)			<0.0001
37+0 to 38+6	1.00	-	
39+0 to 41+6	0.60	0.44–0.81	
Pre-existing maternal condition			0.014
No	1.00	-	
Yes	1.50	1.09–2.06	
Gestational diabetes			0.230
No	1.00	-	
Yes	1.29	0.85–1.94	
Maternal-related pregnancy pathology			0.133
No	1.00	-	
Yes	1.83	0.83–4.02	
Fetal-related pregnancy pathology			0.013
No	1.00	-	
Yes	3.90	1.34–11.37	
Neonatal birth weight			
<3,000 g	1.11	0.78–1.58	0.169
3,000–4,000 g	1.00	-	0.553
≥4,000 g	2.35	1.57–3.53	0.068

The predictors of delivery method for Robson category 2a are presented in Table 3. Women aged 20-29 years had a 30% lower probability of giving birth by CS. Women who smoked had a 91% higher probability of giving birth by CS. Women who underwent assisted reproductive technology had a 51% higher probability of giving birth by CS. Women with gestational diabetes had a 34% lower probability of giving birth by CS. Women whose neonates’ birth weight was less than 3,000 g had a 27% lower probability of giving birth by CS compared to those who gave birth to neonates weighing over 3,000 g. Women whose newborn’s birth weight was ≥4,000 g had three times the probability of giving birth by CS.

**Table 3:** Multiple logistic regression for Robson category 2a. Dependent variable: cesarean versus vaginal birth. P-values were derived from logistic regression. OR = odds ratio; CI = confidence interval

Variable	OR	95% CI	P-value
Age			0.001
20–29 years	0.70	0.54–0.90	0.005
30–39 years	1.00	-	
>40 years	1.68	0.98–2.90	0.059
Ethnicity			<0.0005
Other	1.00	-	
Greek	1.91	1.49–2.44	
Smoking			<0.0005
No	1.00	-	
Yes	1.91	1.49–2.44	
In vitro fertilization			0.044
No	1.00	-	
Yes	1.51	1.01–2.26	
Gestational age (weeks)			0.005
37–38	1.00	-	
39–41	1.44	1.12–1.86	
Pre-existing conditions			0.793
No	1.00	-	
Yes	0.97	0.76–1.24	
Gestational diabetes			0.019
No	1.00	-	
Yes	0.66	0.47–0.93	
Maternal-related pregnancy pathology			0.275
No	1.00	-	
Yes	1.42	0.75–2.68	
Fetal-related pregnancy pathology			0.129
No	1.00	-	
Yes	2.44	0.77–7.73	
Neonatal birth weight			<0.0005
<3,000 g	0.73	0.55–0.96	0.027
3,000–4,000 g	1.00	-	-
≥4,000 g	2.95	1.89–4.12	<0.0005

## Discussion

The Robson classification system is used to categorize and analyze data in clinical audit cycles to reduce CS rates [5]. In this context, the present study aimed to analyze the parameters within the Robson 1 and 2a categories associated with CS. As indicated by the analysis, several clinical factors were associated with CS, reflecting the need to use CS to avoid complications. For example, maternal age was a strong predictor in both groups, in line with the already known impact of age on complications [15], which may indicate a higher need for CS [16]. Hence, the Robson classification serves both as an audit tool and a starting point, from which it is essential to examine further clinical parameters to decide whether to proceed with CS or not.

Despite the obvious need to perform CS according to the risk profile of the women, a careful examination of the predictors in the Robson 2a category is indicative of its excessive use. A first point of concern was nationality, which was associated with a higher probability of CS in the Robson 2a group. In this study, Greek women had a significantly greater chance of undergoing a CS compared to non-Greek women. This may reflect a cultural preference, suggesting that Greek women might be more likely to request a CS than women of other national backgrounds. According to Maffi [17], the perception of whether a CS is considered “natural” is influenced not only by biomedical factors but also by cultural values. Greece has one of the highest CS rates in Europe [7,8], which may have contributed to the normalization of the procedure. As a result, even minor risks during labor may be perceived as a sufficient reason to opt for CS. Although there is no strong evidence supporting the cultural attribution, the high rates of CS in Greece allow the development of a relevant hypothesis. This could explain why the aggravating effect of nationality was observed only among Robson 2a women, and not in Robson 1, where such clinical signals were absent.

Smoking during pregnancy emerged as a statistically significant predictor of CS in our analysis. However, this finding should be interpreted with caution. In contrast to our results, Janoudi et al. [18], analyzing a large Canadian cohort of 134,088 women, found no association between smoking and increased odds of CS delivery. Furthermore, the literature presents a complex picture, with some studies suggesting that smoking may be linked to lower odds of certain complications such as preeclampsia [19], although this remains a debated and controversial issue. Given these inconsistencies and the dose-dependent relationship between smoking and various adverse pregnancy outcomes [20], it is possible that smoking acts as a proxy for other underlying factors rather than exerting a direct causal influence. Therefore, we interpret this association as a potentially misleading indicator rather than a clearly modifiable risk factor, and further research is warranted to clarify the mechanisms involved.

In any case, those indicators should not lead to the conclusion that all clinical factors are misinterpreted. For example, in both categories analyzed, IVF was associated with increased odds for CS. This is also in line with the findings of Janoudi et al. [18], who noted that IVF was a factor associated with increased odds for CS in women classified in Robson 1 and 2a categories. Similarly, Richmond et al. [21] examined a sample of women with infertility in Canada (n = 921,023), reporting that IVF was associated with CS across all Robson categories. Therefore, these potentially misleading indicators do not replace established clinical predictors, but may act as additional, albeit uncertain, contributors to the decision-making process regarding CS.

The aforementioned findings lead to the conclusion that it is necessary to take action to reduce the unjustified CS in low-risk cases in Greece, meaning those classified as Robson 1 and 2a categories. First, it is essential to counter the cultural norms favoring the overuse of CS in Greek society. As the media has a strong impact on shaping cultural norms toward CS [22], it would be reasonable to develop public campaigns informing about the cases where CS is needed. The lack of awareness is a major driver of CS overuse [23]. Therefore, such campaigns would target both the cultural norms promoting CS overuse and the lack of awareness. Such a strategy could have a positive impact on the reasonable use of CS in low-risk cases in Greece.

Second, it is necessary to develop interventions aiming to inform health professionals involved in birth care about the cases where CS is essential. The lack of knowledge among healthcare professionals is a significant problem, leading to the overuse of CS [24]. The results of the present study indicate that such educational interventions have to be specifically targeted to the distinction between the risk accounting for each Robson category and the potential aggravating effect of demographic and clinical factors for women within each of the Robson categories. Such interventions could be extremely helpful to terminate the “one-size-fits-all” solution of CS.

Third, it is important to address the structural problems of the way that private maternal care operates in Greece, which is related to the overuse of CS (e.g., more CS in daytime [25]). Addressing this problem might be much more difficult, as it needs governmental intervention. The Ministry of Health has to set a framework, conduct audits, and develop mechanisms to reduce the excessive use of CS in Greece. This study adds to our previous findings [11] regarding not only the overuse of CS in cases of low risk but also clarifies the cases where wrong signals serve as overjustification of CS. In combination, these two studies indicate the magnitude of the problem, highlighting the need for government intervention and regulation on such a serious public health issue to ensure safety and quality in perinatal health. Previous policies in countries such as Brazil and Iran have primarily focused on incentives [26]. Yet, the magnitude of the problem in Greece is escalating, leading to the conclusion that a stricter regulatory framework from the government is essential.

This study has some limitations that must be reported. First, data collection was conducted in 2019, and some of the findings may not reflect the current situation. This limitation is not related only to the years that have passed but also to some evidence from Greece [27], as well as from other countries [28,29], indicating that inequality in maternity care might have increased compared to the pre-COVID-19 era, leading to different needs and dynamics. Second, the analysis focused on a single private hospital, which may not represent the overall management of Robson 1 and 2a cases in Greece, questioning the generalizability of the results. Third, the absence of control for confounding factors (e.g., body fat apart from body mass index, information for labor management protocols applied) could lead to confounding bias, potentially impacting the observed associations. In addition, generalizability was further constrained by the fact that the study was conducted in a single country, i.e., Greece, which has some of the highest CS rates in Europe [7,8], potentially limiting applicability to countries with different maternity care contexts. Finally, the cultural attribution of the ease in conducting CS is not directly supported by the study’s data, but rather by the authors’ interpretation.

Based on the above, further research should be conducted to gain a broader understanding of the factors contributing to CS in low-risk cases in Greece, as well as in other European countries, especially in those with high rates of CS. To address limitations in generalizability, multicenter studies are highly encouraged. Controlling for confounders is also warranted to depict the impact of clinical and demographic drivers more accurately. In general, further research is essential to identify the factors contributing to CS in the post-pandemic world, which could be somewhat different compared to the previous data.

## Conclusions

The Robson classification serves as an audit tool, from which it is essential to examine further clinical parameters to decide whether to proceed with CS or not. The inclusion of additional clinical and non-clinical parameters enhances evidence-based decision-making on the mode of delivery. Notably, an analysis of factors associated with CS in Robson categories 1 and 2a revealed significant differences. In Robson category 1, women <30 years of age and without pre-existing conditions had significantly lower odds of cesarean section, while smoking, IVF, and fetal pathologies during pregnancy were associated with increased odds of CS. In Robson category 2a, similar trends were observed for age and smoking, with additional significant factors being birth weight and gestational diabetes. These findings underscore the need to develop interventions that improve the quality of perinatal care and promote evidence-based decision-making during childbirth. Future studies that incorporate comparisons between the public and private sectors or assess the impact of policy interventions are needed to further understand and address the phenomenon.
